# Effects of Cognitive Training on Resting-State Functional Connectivity of Default Mode, Salience, and Central Executive Networks

**DOI:** 10.3389/fnagi.2016.00070

**Published:** 2016-04-12

**Authors:** Weifang Cao, Xinyi Cao, Changyue Hou, Ting Li, Yan Cheng, Lijuan Jiang, Cheng Luo, Chunbo Li, Dezhong Yao

**Affiliations:** ^1^Key Laboratory for NeuroInformation of Ministry of Education, Center for Information in Medicine, High-Field Magnetic Resonance Brain Imaging Key Laboratory of Sichuan Province, School of Life Science and Technology, University of Electronic Science and Technology of ChinaChengdu, China; ^2^Shanghai Key Laboratory of Psychotic Disorders, Shanghai Mental Health Center, Shanghai Jiao Tong University School of MedicineShanghai, China; ^3^Shanghai Changning Mental Health CenterShanghai, China; ^4^Bio-X Institutes, Key Laboratory for the Genetics of Developmental and Neuropsychiatric Disorders, Ministry of Education, Shanghai Jiao Tong UniversityShanghai, China; ^5^Brain Science and Technology Research Center, Shanghai Jiao Tong UniversityShanghai, China

**Keywords:** aging, cognitive training, resting-state fMRI, functional connectivity, brain network

## Abstract

Neuroimaging studies have documented that aging can disrupt certain higher cognitive systems such as the default mode network (DMN), the salience network and the central executive network (CEN). The effect of cognitive training on higher cognitive systems remains unclear. This study used a 1-year longitudinal design to explore the cognitive training effect on three higher cognitive networks in healthy older adults. The community-living healthy older adults were divided into two groups: the multi-domain cognitive training group (24 sessions of cognitive training over a 3-months period) and the wait-list control group. All subjects underwent cognitive measurements and resting-state functional magnetic resonance imaging scanning at baseline and at 1 year after the training ended. We examined training-related changes in functional connectivity (FC) within and between three networks. Compared with the baseline, we observed maintained or increased FC within all three networks after training. The scans after training also showed maintained anti-correlation of FC between the DMN and CEN compared to the baseline. These findings demonstrated that cognitive training maintained or improved the functional integration within networks and the coupling between the DMN and CEN in older adults. Our findings suggested that multi-domain cognitive training can mitigate the aging-related dysfunction of higher cognitive networks.

## Introduction

As people age, they experience cognitive decline, which involves working memory, executive function and attention, among other functions, and this occurs with particular frequency in late life, resulting in an increasingly poor quality of life (Hedden and Gabrieli, 2004; Grady, 2012). To postpone the cognitive decline associated with aging, increasing numbers of studies have conducted interventions with older adults, focusing on cognitive training and physical exercise (Park and Bischof, 2013; Tanigawa et al., 2014). Results of neuropsychological studies have demonstrated benefits of cognitive functions after interventions in old adults, including improvements in working memory capacity, reasoning, and processing speed (Neely and Backman, 1995; Oswald et al., 2006; Bamidis et al., 2015; Leung et al., 2015). Our previous study has also demonstrated that the effects of interventions on cognition are maintained at 1 year after the training ended (Cheng et al., 2012). On the other hand, neuroimaging studies have proved that multi-domain training affects the spontaneous brain activity in healthy older adults, for example, altered regional homogeneity of spontaneous fluctuations in temporal gyrus and cerebellum, enhanced amplitude of low frequency fluctuations in frontal gyrus and cerebellum (Yin et al., 2014; Zheng et al., 2015). In another study, improved memory performance during memory task after training has been shown to associate with increased brain activation in the prefrontal, temporal, and parietal area in older adults (Kirchhoff et al., 2012). Also, the reduction in functional connection (FC) between the superior parietal cortex and inferior temporal lobe is observed after training in older adults, which is associated with training success (Strenziok et al., 2014). These findings suggest that the interventions bring about not only enhanced behavioral outcomes but improvement of the brain function in older adults (Kraft, 2012; Bamidis et al., 2014).

Recent studies suggest that functional brain networks, a higher level of organization of brain regions, are needed to better understand improved cognitive function after intervention (Langer et al., 2013; Bamidis et al., 2014). Using functional magnetic resonance imaging (fMRI), more than ten functional brain networks constituted of spatially distinct brain regions have been identified either at specific tasks (Calhoun et al., 2009) or at rest, including the default mode network (DMN), the salience network (SN) and the central executive network (CEN; Smith et al., 2009; Luo et al., 2012). Three (DMN, SN, and CEN) of these stable networks have been considered the ‘core’ neurocognitive networks for understanding higher cognitive functions and have received the most attention in cognitive studies (Sridharan et al., 2008; Bressler and Menon, 2010; Luo et al., 2011, 2014b). The DMN, which includes mainly the posterior cingulate cortex (PCC), medial prefrontal cortex (MPFC) and inferior parietal lobe (IPL), is involved in self-referential and autobiographical memory functions (Raichle et al., 2001; Buckner et al., 2008). The SN includes the bilateral anterior insula (AI) and dorsal anterior cingulate cortex, and it is associated with detecting internal and external events salient to homeostasis. In contrast, the CEN, composed of the dorsolateral frontal and parietal neocortices, is engaged in cognitively demanding tasks, such as decision making and working memory (Seeley et al., 2007). Many studies have indicated that these three networks, along with the disruption of FC within network and the anti-correlation between the DMN and CEN, are strongly affected by normal aging and Alzheimer’s disease, which is associated with cognitive impairments (Meunier et al., 2009; Wu et al., 2011; Onoda et al., 2012; Tomasi and Volkow, 2012; Cao et al., 2014).

Previous studies have shown that brain networks are affected by interventions, such as the DMN and CEN, associated with improved behavioral outcomes and cognitive performances (Patel et al., 2013; Bamidis et al., 2014; Luo et al., 2014a; Tang and Posner, 2014). For instance, intervention studies, including either cognitive or physical training, reveal changes in the resting-state FC within the DMN and CEN in healthy adults (Voss et al., 2010, 2013; Taylor et al., 2013). Interventions have also been conducted on older adults to investigate the changes in brain networks that subserve cognitive function (Voss et al., 2010; Bamidis et al., 2014; Luo et al., 2016). A study of multimodal intervention in older adults has shown improved resting-state FC between the MPFC and medial temporal lobe in the DMN after training, suggesting an association with better cognitive performance (Li et al., 2014). Increases in cerebral blood flow and resting-state FC within the DMN and CEN are also observed after complex cognitive training in healthy seniors (Chapman et al., 2015). In addition, a study also demonstrated the decreased FC between the DMN and CEN after working memory training in health adults (Takeuchi et al., 2013). These findings suggest that training can affect FC of certain brain networks, such as DMN, CEN. However, no study has investigated the effects of multi-domain cognitive training on resting-state FC within and among the three networks (DMN, SN, and CEN) in older adults.

The purpose of this study is to explore the effects of cognitive training on resting-state FCs within and between the three networks in old adults. We hypothesized that multi-domain cognitive training would result in changes of resting-state FC within and among the three networks in healthy older adults, which might be beneficial for counteracting decreased integration within network and impaired coupling between networks with aging. In our work, we investigated the resting-state FC of three networks using seed-based FC analysis at baseline and at 1 year after training ended. The relationship between cognitive improvements and the changes of FC was also analyzed.

## Materials and Methods

### Participants

The current work included 40 healthy older adults from two randomized groups [the multi-domain training group (*n* = 23) and the control group (*n* = 17)], who were recruited through a dispatched notice/broadcasting by the local institute of community service in three community centers around Tongji Hospital in Shanghai from March 2008 to April 2008. After a personal interview with professional interviewers, all participants then underwent cognitive measurements and fMRI scanning. The inclusion criteria included the following: age (65–75 years); educational year (more than 1 year); normal hearing, vision, or communication status; normal functional capacity; living independently in the community; score on the Chinese version of the Mini-Mental State Examination (MMSE) of 19 or above [the lower cut-off point of the MMSE score is due to the lower educational level in China (Li et al., 2006)]; and being free of neurological or psychiatric disorders and of brain injury. Exclusion criteria included the following: obvious cognitive decline, a diagnosis of AD; serious neurological or psychiatric disorders such as major depressive disorder, brain cancer, and schizophrenia. One left-handed subject in the multi-domain training group was included due to the absence of a relationship between the training-related changes and handedness (Mackey et al., 2013). This study was approved by the Ethical Committee of the East China Normal University. All participants gave written informed consent, and they were not provided monetary compensation for their participation in this study.

### Neuropsychological Measurements

#### Interventions

Cognitive training employed a randomized, controlled design. The multi-domain training group was divided into a small group, and the training procedure took place in a classroom in Tongji Hospital. All participants received 24 sessions of cognitive training over a 3-months period at a frequency of twice a week. The multi-domain cognitive training targeted memory, reasoning, and problem-solving strategies using approaches such as visual-spatial map reading, handcraft making, healthy living, and physical exercise. Each session lasted 60 min. A lecture about a common disease in aging people was presented during the first 15 min of each session. Then, the trainer taught the participants about a special cognitive strategy or technique via slide lecture during the second 30 min. The newly practiced skills were consolidated by dealing with some real-life problems during the last 15 min. The wait-list control group served as a match for the social contact associated with cognitive training. The multi-domain training group and the control group attended a lecture about healthy living every 2 months. More details about the training are provided in our previous study (Cheng et al., 2012).

#### Cognitive Measurements

To evaluate the effects of intervention on cognitive function, composite outcome measurements were created, including the Repeatable Battery for the Assessment of Neuropsychological Status (RBANS, Form A), which shows good validity and reliability in a Chinese community-living elderly sample (Lim et al., 2010; Cheng et al., 2011), the trail-making test (TMT; Ashendorf et al., 2008), the visual reasoning test (Xiao et al., 2002) and the Color Word Stroop test (CWST; van Boxtel et al., 2001). All measurements were conducted at baseline and at 1 year after training.

To examine the effect of cognitive training on cognition, we compared the pre- and post-training changes between the multi-domain cognitive group and the control group using one-way analyses of covariance (ANCOVAs) with the differences between the 1-year post-test and baseline measurements as dependent variables and scores at baseline as covariates to exclude the possibility that any pre-existing difference in the measures between the groups affected the results of each measure (*p* < 0.05). In addition, two-sample *t*-tests were applied to investigate the differences in cognition measurements at baseline between the two groups. The confounding covariates, including age, gender and education years, were regressed in all statistical analyses.

### Data Acquisition

All functional and structural imaging data were acquired using a Siemens 3T MRI scanner (Erlangen, Germany) at East China Normal University, Shanghai, China. All participants underwent scanning twice, at baseline and at 1 year after training. To minimize head motion, we fixed the subjects’ heads using foam pads. Resting-state functional images were collected using a single-shot, gradient-recalled echo planar imaging sequence [repetition time (TR) = 2000 ms, echo time (TE) = 25 ms and flip angle (FA) = 90°, field of view (FOV) = 240 mm × 240 mm, in-plane matrix = 64 × 64, slice thickness = 5 mm, voxel size = 3.75 mm × 3.75 mm × 5 mm], generating 32 slices. The functional scanning lasted for 310 s, yielding a total of 155 volumes. To ensure magnetic field stabilization, the first five volumes were discarded. During the resting-state scanning, the subjects were asked to lie with their eyes closed, not to fall asleep, and not to think of anything in particular. Axial T1-weighted anatomical images were acquired using a magnetisation-prepared rapid gradient-echo sequence, generating 160 slices (TR = 1900 ms, TE = 3.43 ms, FA = 90°, matrix size = 256 × 256, FOV = 240 mm × 240 mm, slice thickness = 1 mm, voxel size = 0.9375 mm × 0.9375 mm × 1 mm).

### Data Preprocessing

#### Resting-State fMRI Data

Imaging data preprocessing was performed using the SPM8 software package^1^. First, all imaging data were corrected for the slice-timing and realigned for head movement correction. Accounting for the effect of head motion on the FC analysis, we excluded all the participants whose head movement exceeded 1 mm or 1°. Furthermore, we also calculated the framewise displacement (FD) given by

(1) $$FD_{i}=\left|\Delta d_{ix}\right|+\left|\Delta d_{iy}\right|+\left|\Delta d_{iz}\right|+\left|\Delta \alpha_{i}\right|+\left|\Delta \beta_{i}\right|+\left|\Delta \gamma_{i}\right|,$$

where *i* is the *i*-th time point and

(2) $$\Delta d_{ix}=d_{(i-1)x}-d_{ix}(similarlyford_{iy},d_{iz},\alpha_{i},\beta_{i},\gamma_{i}).$$

The FD_0_ was set to zero and rotational displacements were converted from degrees to millimeters (Power et al., 2012). Then, the resulting functional data were normalized to the Montreal Neurological Institute (MNI) template using a 12-parameter affine transformation (resampled to 3 mm × 3 mm × 3 mm). Finally, the imaging data were spatially smoothed with a 6-mm full-width at half maximum (FWHM) Gaussian kernel. We applied a temporal band-pass filter (the frequency range of 0.01 and 0.08 Hz) to reduce the low frequency drift and high frequency physiological noise (Fox et al., 2005; Jiang et al., 2015; Luo et al., 2015). Several nuisance covariates including white matter (WM) signal, cerebrospinal fluid (CSF) signal, global signal, and six head motion parameters were also removed from the time course of all brain voxels using a multiple linear regression analysis.

#### Preprocessing of T1-Weighted Images

Previous studies provided the evidence of the loss of gray matter volume (GMV) with aging and of the potential effects on the functional results (Damoiseaux et al., 2008). In the present study, we increased the reliability of resting-state fMRI analysis by controlling for the GMV. The T1-weighted images were preprocessed using the voxel-based morphometry (VBM8^2^) toolbox in SPM8. The native T1-weighted image was normalized to MNI space using an exponentiated lie algebra (DARTEL) approach. The resulting images were then segmented into gray matter (GM), WM and CSF. For each subject, the GMV of the whole brain was calculated.

#### Functional Connectivity Analysis

To evaluate our hypothesis, a seed-based FC analysis was performed to examine the training effect on FC within and between the DMN, SN, and CEN. Three seeds were selected based on previous studies: the PCC (0, -52, 20), the right AI (38, 26, -10) and the right dorsolateral prefrontal cortex [right DLPFC (44, 36, 20)] to constitute the DMN, SN, and CEN, respectively (Fransson, 2006; Seeley et al., 2007; Luo et al., 2011). The average signal of spherical regions with nine-millimeter diameter centered at the three seeds was extracted. The FC map for each network was obtained by calculating Pearson correlation coefficient between the average time course of seed and each voxel in the whole brain for each subject. A Fisher z-transformation was used to convert the correlation coefficient of each voxel to a normal distribution (Shao et al., 2012; Chen et al., 2015). Thus, the individual z-score maps were obtained for each seed and subject. A one-sample *t*-test was used to identify the within-group FC map for each network. The significance level was set at *p* < 0.05 (FDR-corrected, cluster size > 621 mm^3^).

#### Statistical Analysis

To evaluate the training effect on FC within and between the three networks, a whole-brain voxel-wise 2 (between-subject factor: training and control groups) × 2 (within-subject factor: baseline and 1-year after training ending) repeated analysis of variance (ANOVA) was performed using the explicit mask from the union set of the one-sample *t*-test results of three networks, by controlling for the whole brain GMV, as well as age, gender, education years and FD as confounding covariates. This analysis was performed using SPM8, and the significance level was set at *p* < 0.05 (cluster size > 621 mm^3^). *Post hoc* comparisons (paired *t*-tests) were carried out for each significant interaction effect to clarify the effect of training on FC for each group.

#### Neural Correlate Analysis

To explore the relationship between FC and cognitive measurements, z-transformed connectivity values were extracted from regions that showed significant training-related effect, and the difference between the baseline and post-test was computed. Partial correlations were performed between the changes of resting-state FC and the pre- and post-training differences in the scores on the cognitive measures, while controlling for age, gender, and education years.

## Results

### Demographic and Neuropsychological Tests

At baseline, 23 subjects (one left-handed) from the multi-domain training group and 17 subjects from the control group underwent cognitive measurements and the fMRI scanning. Eighteen subjects in the multi-domain training group and 14 in the control group finished both the cognitive measurements and the fMRI scanning at 1 year after the intervention, and they were involved in the final analysis. A total of eight participants withdrew (one death, two cancers, one operation, and four rejecting scanning). No significant differences in age, gender, education years, FD and MMSE score were found between the multi-domain training group and the control group (*p* > 0.05; **Table 1**).

**Table 1:** Demographic information about the subjects at different timepoints.

		Multi-domain training group	Control group	*p*
Age (year)	Baseline	70.61 ± 3.29	68.59 ± 3.24	0.838
(mean ± SD)	One-year post-test	72.39 ± 3.43	70.85 ± 4.05	0.782
Gender (male)	Baseline	23 (16)	17 (9)	0.283
	One-year post-test	18 (13)	14 (9)	0.631
Education (year)	Baseline	10.91 ± 3.65	10.64 ± 3.06	0.452
(mean ± SD)	One-year post-test	11.11 ± 4.25	10.09 ± 3.34	0.383
MMSE	Baseline	27.57 ± 2.57	28.17 ± 1.94	0.505
(mean ± SD)	One-year post-test	27.72 ± 2.16	27.85 ± 2.31	0.900
FD	Baseline	0.12 ± 0.04	0.14 ± 0.05	0.448
(mean ± SD)	One-year post-test	0.12 ± 0.04	0.16 ± 0.07	0.068

*FD, Framewise displacement; MMSE, the Mini-Mental State Examination; SD, standard deviation.*

No significant differences in all cognitive measurements were found at baseline between the two groups (*p* > 0.05; **Table 2**). However, the multi-domain training group showed significant improvements in performance on language (*p* = 0.036) and delayed memory (*p* = 0.027) and marginally significant improvement on the RBANS total score (*p* = 0.058) at 1-year after training compared to the baseline (**Table 2**).

**Table 2:** Baseline and 1-year post-test scores for psychological measures (mean ± SD).

	Multi-domain training		Contorl			
	Baseline	One-year Post-test	Baseline	One-year Post-test	*P*^a^	*P*^b^
RBANS total score	93.17 ± 16.10	106.94 ± 12.90	93.07 ± 15.00	99.57 ± 13.75	0.058	0.971
Immediate memory	86.22 ± 16.45	103.17 ± 23.35	84.00 ± 14.34	100.5 ± 14.33	0.695	0.694
Visuospatial	106.78 ± 12.86	103.9 ± 11.56	106.86 ± 19.05	103.00 ± 16.81	0.981	0.922
Language	92.44 ± 12.92	101.00 ± 8.39	93.29 ± 7.12	95.86 ± 5.86	**0.036^∗^**	0.861
Attention	90.67 ± 19.39	94.94 ± 15.17	91.5 ± 15.25	88.93 ± 15.24	0.22	0.776
Delayed memory	98.28 ± 18.86	118.50 ± 12.41	99.00 ± 13.13	110.21 ± 14.80	**0.027^∗^**	0.999
The CWST						
Color interfere	20.16 ± 13.95	27.33 ± 17.01	14.57 ± 7.62	23.85 ± 14.33	0.884	0.125
Word interfere	38.61 ± 15.56	39.89 ± 17.35	39.71 ± 14.10	42.00 ± 19.00	0.669	0.642
The Visual Reasoning Test	5.78 ± 1.35	6.56 ± 1.89	5.86 ± 2.74	5.93 ± 2.46	0.169	0.894
The TMT						
Trail A complete time	79.50 ± 23.60	73.72 ± 33.93	87.21 ± 37.37	77.07 ± 29.22	0.739	0.381
Trail B complete time	157.89 ± 55.41	138.5 ± 48.27	153.5 ± 68.11	142.57 ± 49.6	0.634	0.830

*^∗^p<0.05. CWST, Color Word Stroop test; RBANS, Repeatable Battery for the Assessment of Neuropsychological Status; TMT, Trail Making test. ^a^One-way ANCOVAs with differences between 1-year post-test and baseline in psychological measures as dependent variables, by controlling for scores of the psychological measures at baseline, age, gender and education years as covariates. ^b^Two sample *t*-test with scores of the psychological measures at baseline as dependent variables, by controlling for age, gender, and education years as covariates.*

### The Effect of Cognitive Training on Networks

Three networks were constituted by seed-based FC analysis, seeding at the PCC for the DMN, at the right AI for the SN and at the right DLPFC for the CEN. The DMN mainly involved the PCC/precuneus, VMPFC, DMPFC, bilateral IPL, bilateral temporal lobe, cerebellar lobule IX and crus II (Supplementary Figure S1). The SN mainly included the bilateral AI, DMPFC, dACC, and bilateral TPJ (Supplementary Figure S2), and the CEN was composed of the DLPFC, DMPFC, and IPL, all bilaterally (Supplementary Figure S3).

The 2 × 2 repeated measure ANOVA revealed significant interactions (*p* < 0.05) for FC within and between networks. In this study, this analysis was performed for the FCs that showed no significant difference between the training and control groups at baseline, because a training-related effect was the only possibility.

**FIGURE 1:**
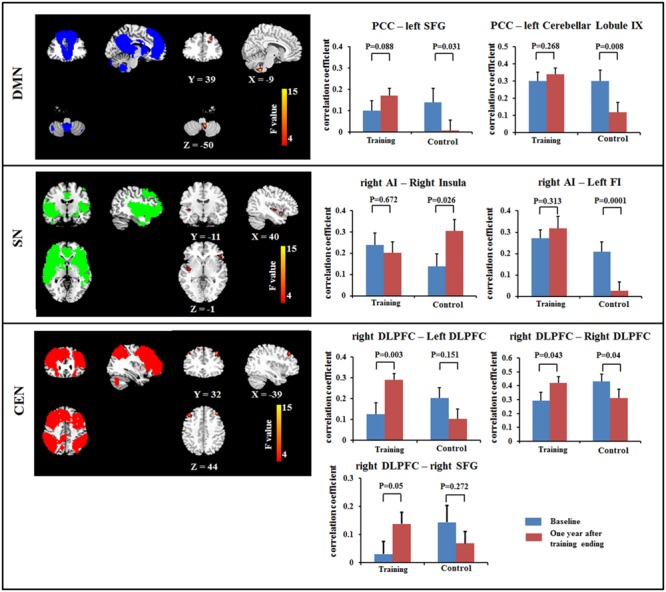
**Results of Resting-state functional connectivity (FC) within the DMN (top), within the SN (middle) and within the CEN (bottom) using a voxel-based 2 × 2 repeated ANOVA analysis (*p* < 0.05, cluster size > 621 mm^3^).** Bars at the right show the mean correlation coefficient of significantly altered FC within networks in training and control groups at the baseline (blue) and 1-year after training ended (red). CEN, Central executive network; DMN, default mode network; DLPFC, dorsolateral prefrontal cortex; FI, frontoinsula; PCC, posterior cingulate cortex; SFG, superior frontal gyrus; SN, salience network. The left side of the image corresponds to the right side of the subject.

The ANOVA revealed the significant interactions for the FC within the DMN [PCC – left superior frontal gyrus (SFG) and PCC – cerebellar lobule IX]; within the SN (right AI – right middle/posterior insula, and right AI – left frontoinsula) and within the CEN (right DLPFC – bilateral DLPFC and right DLPFC – right SFG). *Post hoc* comparison demonstrated that some connections showed increased or maintained FC in the training group but decreased FC in the control group, including those between the PCC and the left SFG and between the PCC and cerebellar lobule IX within the DMN, between the right AI and left frontoinsula within the SN, and between the right DLPFC and the bilateral DLPFC and between the right DLPFC and the right SFG within the CEN (**Figure 1**; **Table 3**). In addition, we observed increased FC between the right AI and right middle/posterior insula in the control group but maintained FC after training in the training group (**Figure 1**; **Table 3**).

**Table 3:** Brain areas showing significant interactions for resting-state functional connectivity using a 2 (between-subject factor: training and control groups) × 2 (within-subject factor: baseline and 1-year after training ended) repeated ANOVA.

Seed	Brain region	BA	MNI coordinate			*F*-score	Cluster size (mm^3^)
			*X*	*Y*	*Z*		
PCC	Left dorsolateral prefrontal lobe	20	-33	15	24	18.64	2187
	Left cerebellar lobule IX		-6	-51	-54	16.2	2241
	Right putamen	9	18	15	-9	13.17	1377
	Right dorsolateral prefrontal lobe		51	0	30	12.86	1782
	Left superior frontal gyrus	20	-24	36	39	11.66	1323
Right AI	Right insula	45	30	-9	-18	15.82	4644
	Left frontoinsula	45	-51	33	-12	11.67	2538
Right DLPFC	Right superior frontal cortex	11	15	27	54	13.30	3186
	Left dorsolateral prefrontal cortex	9	-39	30	42	12.78	1647
	Left inferior parietal lobe		-30	-63	30	13	1431
	Right superior frontal gyrus		42	27	42	12.12	2970

*BA, Broadmann area; CEN, central executive network; DMN, default mode network; SN, salience network; DLPFC, dorsolateral prefrontal cortex; AI, anterior insula; PCC, posterior cingulate cortex.*

**FIGURE 2:**
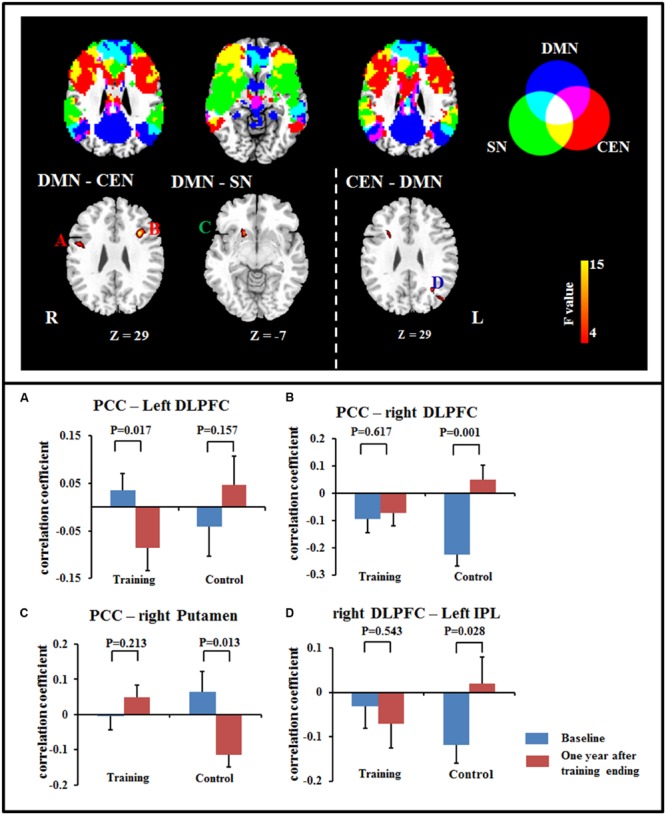
**Significant differences in FC were observed between DMN and CEN **(A,B)**, between DMN and SN **(C)**, and between CEN and DMN **(D)** using a voxel-based 2 × 2 repeated measure ANOVA (*p* < 0.05, cluster size > 621 mm^3^).** Bars at the bottom show the mean correlation coefficient of significant altered FC between networks in training and control groups at the baseline (blue) and 1-year after training ended (red). CEN, Central executive network; DMN, default mode network; DLPFC, dorsolateral prefrontal cortex; IPL, inferior parietal lobe; PCC, posterior cingulate cortex; SN, salience network.

Our results also revealed significant interactions in the FCs between networks, including between DMN and CEN and between DMN and SN. *Post hoc* comparisons showed that the anti-correlations between the DMN and CEN were maintained or increased after training, including an increase in the anti-correlation between the PCC and the left DLPFC in the training group, while this correlation was maintained in control group; meanwhile, the anti-correlation between the PCC and the right DLPFC was maintained in training group and decreased in the control group. For the seed at the right DLPFC, the anti-correlation between the right DLPFC and left IPL was maintained in the training group and increased in the control group. For the connection between the DMN and SN, the connectivity between the PCC and the right putamen was maintained in the training group and decreased in the control group (**Figure 2**; **Table 3**).

### The Correlation between Cognitive Measures and Resting-State fMRI

**FIGURE 3:**
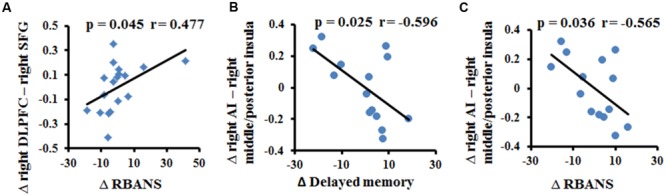
**Correlation between the change in resting-state FC and the change in cognitive performance in the multi-domain training group **(A)** and in the control group (B,C)**.

We found a significant relationship between the changes in FC (between the two fMRI sessions) and the improvements in cognitive performances (*p* < 0.05, uncorrected). As shown in **Figure 3**, the increased FC between the right DLPFC and the right SFG had a slim positive correlation with the RBANS test scores in the multi-domain training group (**Figure 3A**). The increased FC between the right AI and the right middle/posterior insula predicted poorer delayed memory and RBANS scores in the control group (**Figures 3B,C**).

## Discussion

In the present study, we used resting-state fMRI analysis to examine the effects of multi-domain cognitive training on FC within and among three networks (DMN, SN, and CEN). Consistent with our *a priori hypothesis*, changes in resting-state FC within and among the three networks were found at the 1-year after multi-domain cognitive training ended in training group compared to the wait-list control group. After training, the maintained or increased anterior–posterior and interhemispheric FCs within network, including PCC – left SFG (DMN), PCC – cerebellar lobule IX (DMN), right AI – left AI (SN), and right DLPFC – left DLPFC (CEN), were observed in the training group, while these FCs decreased in the control group. Compared to the control group, the training group showed increased or maintained anti-correlated FC between DMN and CEN (PCC – DLPFC and DLPFC – IPL). The coupling between DMN and SN (PCC – putamen) was also maintained in training group, but decreased in the control group. These findings suggested that multi-domain cognitive training might counteract decreased integration within network and impaired coupling between networks with aging.

### Training Effects on FC within Networks

This study found training-related enhanced integration of FC within the DMN, SN, and CEN. In particular, we observed that FCs between PCC and SFG and between PCC and cerebellar lobule IX were maintained in the multi-domain training group, but decreased in the control group. Previous studies have provided consistent evidence that healthy aging is accompanied by decreased FC within the DMN, particularly an anterior–posterior disruption of FC (Andrews-Hanna et al., 2007; Mevel et al., 2013). The decreased FC between the PCC and the SFG in the control group was consistent with previous findings, highlighting age-related disruption of connectivity at rest along the anterior–posterior axis of the DMN (Andrews-Hanna et al., 2007). The role of the cerebellum in cognitive function has been accepted. For example, lobule IX has been mentioned in association with DMN (Habas et al., 2009), and the FC between DMN nodes (PCC and MPFC) and cerebellar regions has been described as modified by aging and by diseases such as dementia (Bernard et al., 2013; Guo et al., 2015). Unlike the decreased FC with normal aging in the control group, multi-domain cognitive training maintained these FCs from the PCC to the SFG and to lobule IX of the cerebellum, suggesting that cognitive training might resist or postpone age-related disruption of FC within the DMN. In the CEN, we also observed increased FC between the right DLPFC and the frontal lobe (bilateral DLPFC and right SFG) at 1 year after training compared with baseline in the multi-domain training group, while these FCs decreased in the control group. These frontal regions have been found to be involved in executive and cognitive function (Ridderinkhof et al., 2004; Grady, 2012), such as working memory, and to be activated by tasks related to these function when the age-related effects were investigated (Grady, 2012). The frontal lobe is known to be affected by training (Park and Bischof, 2013). In addition, the change of FC between the right DLPFC and the right SFG was slight positively correlated with the improvement on the RBANS test in the training group. Thus, we presumed that the multi-domain training might have positive effects on improvement of behavior via enhancing the FC. However, a larger sample size should be considered to verify the relationship between the FC and cognitive function. A recent study of complex cognitive training in healthy seniors also found increased resting-state FC of the DMN and CEN, as well as increased cerebral blood flow (Chapman et al., 2015). Consistent with those reports, our results showed changes in intranetwork FC in the DMN and CEN after cognitive training. These findings might suggest that the cognitive training enhanced integration of FC within networks, resulting in the improvement of cognitive function.

Recent studies demonstrated that the decreased FC within the SN is an important feature of normal aging and is associated with the cognitive impairments (Onoda et al., 2012; He et al., 2013). Consistent with previous studies, we found decreased FC between the right AI and the left frontoinsula in the control group between the two timepoints scanned. However, the FC was maintained in the training group, as indicated by the absence of a difference between the FC values from before and after multi-domain cognitive training. In addition, we observed the FC between the right AI and right middle/posterior insula was maintained in the training group, but increase in the control group. The right AI, as a key node of the SN, is involved in cognitive function; by contrast, the middle/posterior insula serves in sensorimotor functions (Kurth et al., 2010). The increased FC between the right AI and right middle/posterior insula might indicate the disrupted function of the insula, likely reflecting an association with the cognitive decline in old adults. Here, we observed the negative correlation between the increase in the FC between the right anterior and right middle/posterior insula and the improvement of delayed memory and RBANS scores in the control group, probably implicating the relationship between the FC within the SN and the cognition. Thus, we speculated that cognitive training enhanced integration of the SN to retain at least some of the cognitive performance.

### Training-Related Changes of FC between Networks

The triple network model of the DMN, SN, and CEN has been used to understand cognitive dysfunction in neurological and psychiatric disorders (Menon, 2011). A previous study provided evidence for a decrease in the anti-correlation between DMN and CEN in normal aging (Wu et al., 2011). Here, we found that the aging-sensitive anti-correlation between DMN and CEN was maintained after training. A higher anti-correlation between DMN and CEN was also found after working memory training in healthy adults (Takeuchi et al., 2013). In addition, the disruption of SN and DMN connectivity has been observed in dementia (Zhou et al., 2010). Here, the connection between the DMN and SN was maintained in the training group, but decreased in the control group. Our results might also suggest that the anti-correlation of DMN and CEN is relatively sensitive to cognitive training.

### Limitations

Interpreting the present findings requires the consideration of a few key limitations. First, our demographic characteristics, such as sample size and educational level, might affect the generalisability of the results. A larger sample size and a more consistent educational level among the subjects are necessary to verify the effect of cognitive training on the resting-state FC of three networks. Second, because the imaging data were not acquired at intermediate stages and or immediately after the completion of training completion, the effects of training on FCs within and between networks at additional intermediate timepoints and immediately after the completion of training are not studied. Third, we considered the effects of brain atrophy on FC in this work. However, the other focal damage (i.e., vascular lesions) is prevalent in the population and may have contributed to our findings. Fourth, the effects of cognitive training on resting-state FCs of three networks were observed in our study. This result suggested that the training was valid in the current population; however, further work should be performed to verify these conclusions in other populations. Finally, another control group of younger subjects should be considered in future work to determine whether the effects of cognitive training are specific to older adults.

## Conclusion

We found changes in resting-state FC within networks and between the three networks at 1 year post-training, perhaps reflecting that cognitive training enhanced integration within network and maintenance of segregation between DMN and CEN. Our findings provide evidence that multi-domain cognitive training could mitigate the age-related functional alterations involving DMN, SN and CEN, thereby helping older adults maintain brain health.

## Author Contributions

Conceived and designed the work: WC, Cheng Luo, Chunbo Li, DY. Acquired the data: YC, XC, TL, LJ. Analyzed the data: WC, CH, XC, YC. Wrote the paper: WC, Cheng Luo. All authors revised the work for important intellectual content. All of the authors have read and approved the manuscript.

## Conflict of Interest Statement

The authors declare that the research was conducted in the absence of any commercial or financial relationships that could be construed as a potential conflict of interest.

## Footnotes

1 http://www.fil.ion.ucl.ac.uk/spm/

2 http://dbm.neuro.uni-jena.de/
