# Supplementary material for: Effects of Cognitive Training on Resting-State Functional Connectivity of Default Mode, Salience, and Central Executive Networks
Source: Front Aging Neurosci. 2016 Apr 12;8:70. doi: 10.3389/fnagi.2016.00070 (PMC4828428; doi:10.3389/fnagi.2016.00070)
Supplement: Supplementary file 1 [file Data_Sheet_1.DOCX]

## Supplementary Material


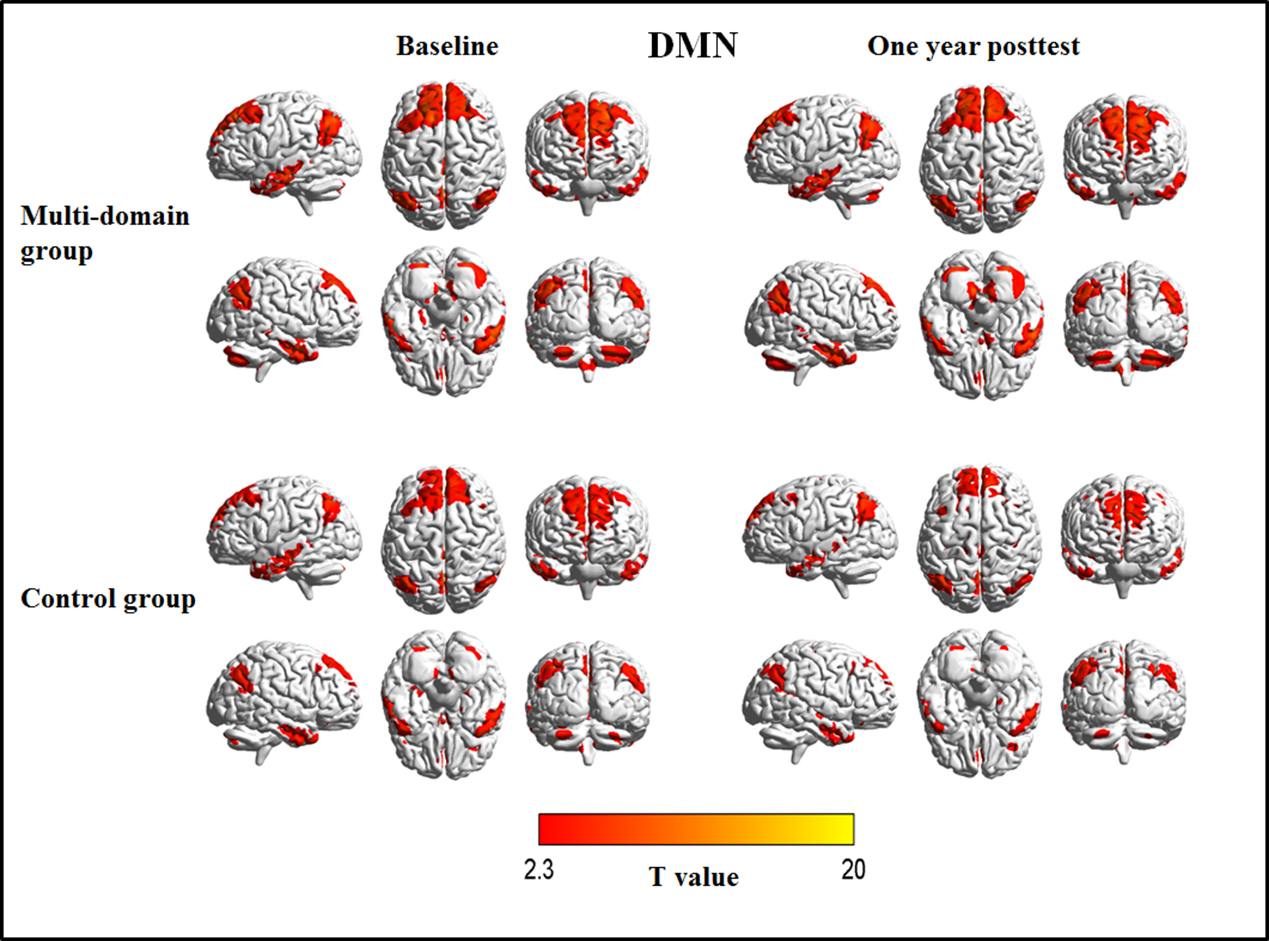


**Figure S1.** Positive FC Maps of DMN in multi-domain training group and the control group at baseline and at one year after training ending respectively. The statistical threshold was P < 0.05 (FDR-corrected, cluster size > 621 mm3).

**
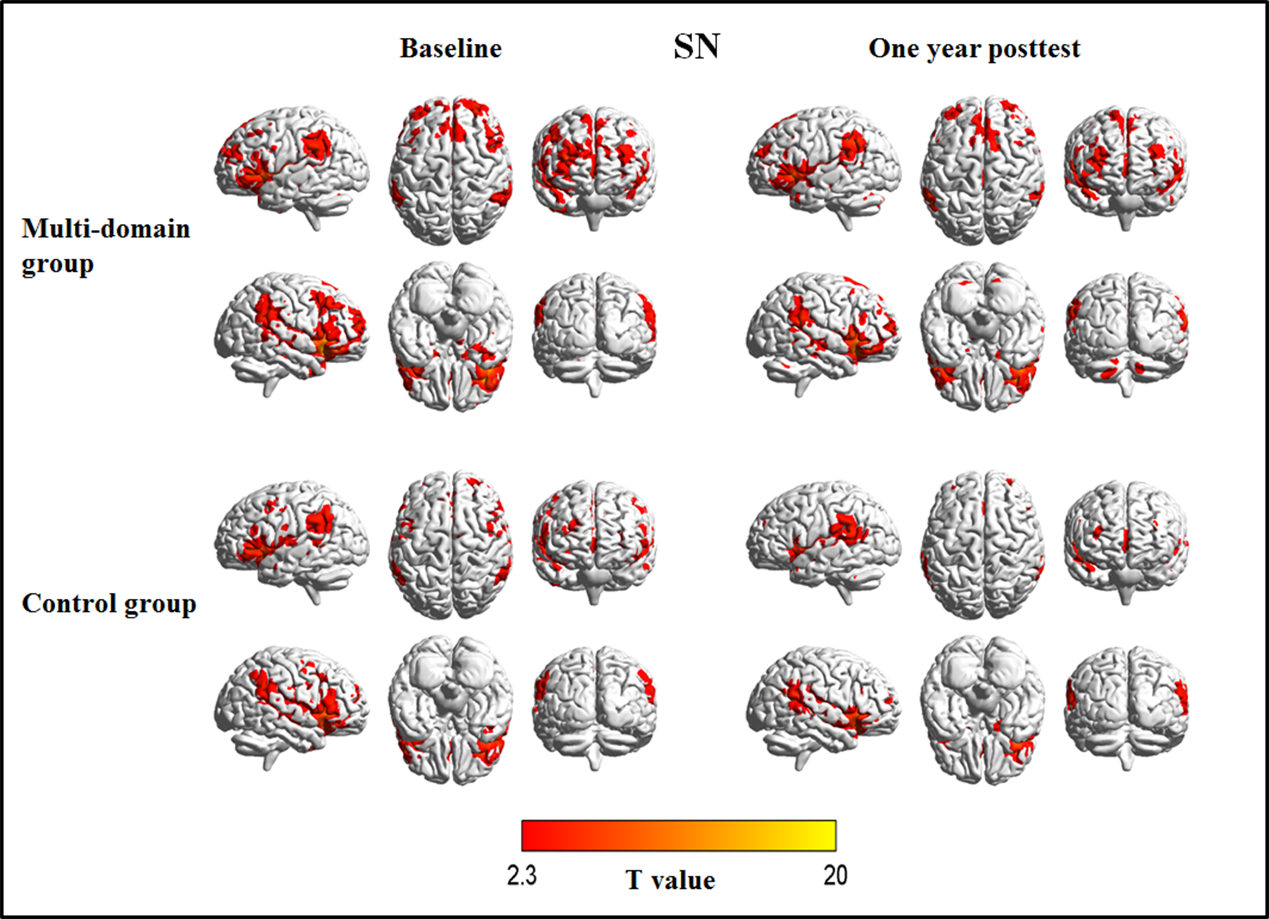
**

**Figure S2.** Positive FC Maps of SN in multi-domain training group and the control group at baseline and at one year after training ending respectively. The statistical threshold was P < 0.05 (FDR-corrected, cluster size > 621 mm3).

**
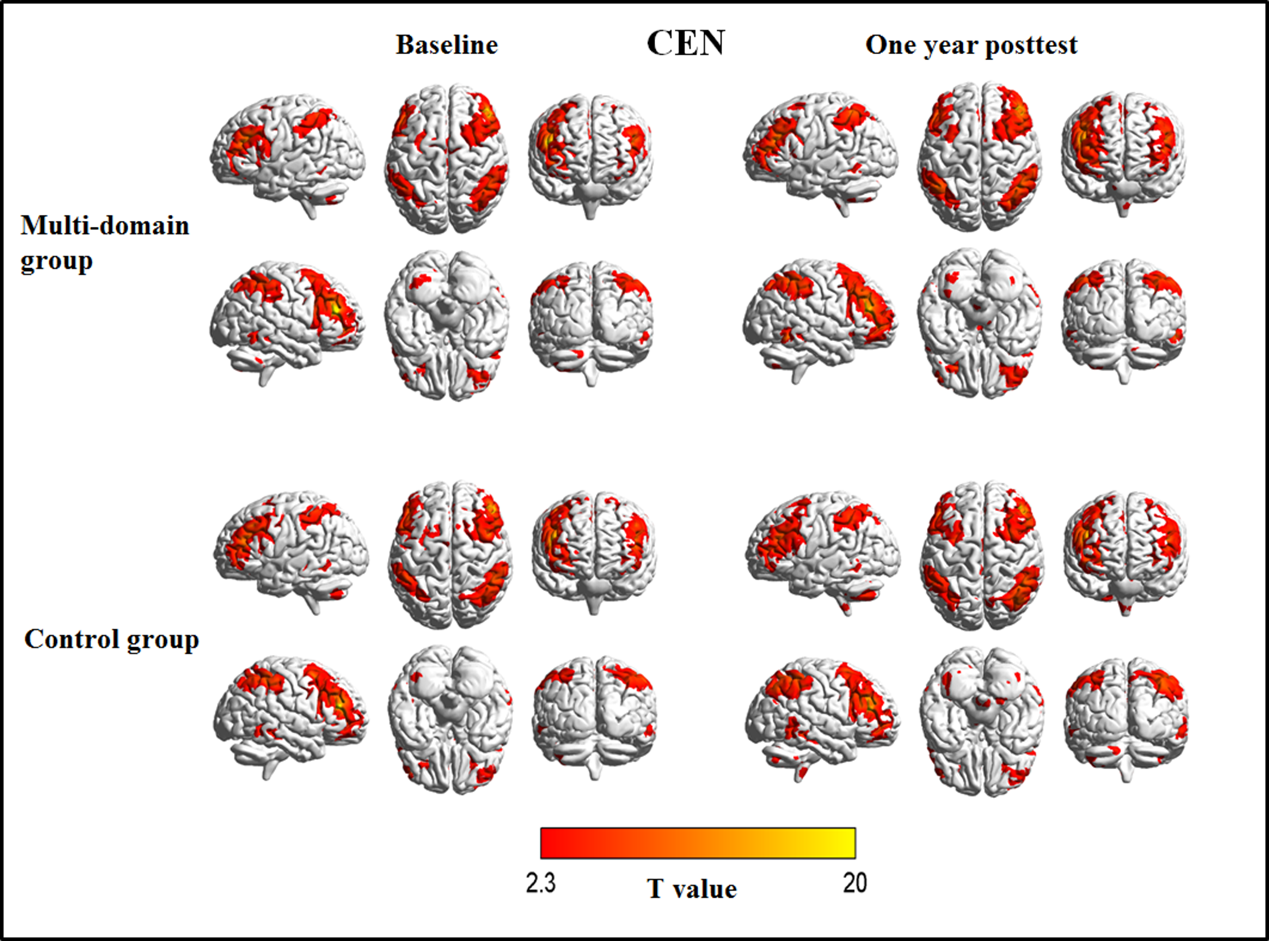
**

**Figure S3.** Positive FC Maps of CEN in multi-domain training group and the control group at baseline and at one year after training ending respectively. The statistical threshold was P < 0.05 (FDR-corrected, cluster size > 621 mm3).
